# A glimpse into the future of genome-enabled plant biology from the shores of Cold Spring Harbor

**DOI:** 10.1186/s13059-016-0870-y

**Published:** 2016-01-11

**Authors:** Seung Y. Rhee, Jane E. Parker, Todd C. Mockler

**Affiliations:** Department of Plant Biology, Carnegie Institution for Science, Panama Street, Stanford, CA 94305 USA; Department of Plant-Microbe Interactions, Max-Planck Institute for Plant Breeding Research, Carl-von-Linné weg, 50829 Cologne, Germany; Donald Danforth Plant Science Center, St. Louis, MO 63132 USA

## Abstract

A report on the 10^th^ plant genome meeting entitled ‘Plant genomes and biotechnology: from genes to networks’, held at Cold Spring Harbor Laboratory, 2–5 December, 2015.

## Introduction

The 10^th^ plant genomics meeting at Cold Spring Harbor Laboratory demonstrated the power of genome-enabled plant biology in a broad spectrum of areas, ranging from environmental adaptation to developmental network modeling and crop improvement. Participants and presenters were a healthy mix of junior and established scientists (47 % of participants being graduate students and postdocs), staying true to the vision of the first meeting in 1997 and the spirit of Cold Spring Harbor Laboratory. The breadth and depth of exciting, unpublished work towards discovering fundamental principles and direct applications in agriculture is impossible to capture in a short meeting report. Therefore, here we highlight some transformative areas whose potential impact is likely to reach beyond plant biology.

## Disruptive tools

A major theme across the sessions was the development of disruptive tools and their application to address technical challenges and to answer key biological questions. Ronan C. O'Malley (Salk Institute, USA) described DNA affinity purification sequencing (DAP-seq), a method he pioneered in the Ecker laboratory and is using to create an atlas of Arabidopsis transcription factor (TF) binding sequences. DAP-seq involves capturing and sequencing genomic DNA fragments using a tagged TF bound to an affinity bead. O'Malley subjected all ~1800 Arabidopsis TFs to DAP-seq and identified motifs for >500 TFs. Interestingly, 60 TFs capture most motif types because members of TF families tend to bind to similar motifs. He also reported that DNA methylation within a TF-binding motif alters binding of 76 % of TFs, with the majority (72 %) being inhibited by methylated sites. Future directions include double DAP-seq or sequential DAP-seq to identify more biologically relevant complex binding sites and to elucidate mechanisms of combinatorial control of gene expression.

Robert VanBuren (a postdoc in the Mockler lab, Donald Danforth Plant Science Center, USA) described the single-molecule genome sequencing of the desiccation-tolerant grass *Oropetium thomaeum*. Resurrection plants such as *O. thomaeum* and *Selaginella lepidophylla* can lose most of their cellular water content during desiccation but ‘come back to life’ upon re-watering. Hence, the genomic resources of these plants have the potential to shed light on mechanisms of stress tolerance. *O. thomaeum* has the smallest known genome among the grasses (~250 Mbp), and was sequenced at ~65× coverage using PacBio’s P6-C4 chemistry making it the first published genome sequenced using single-molecule sequencing. The exceptional quality of the resulting genome assembly was enabled by high-quality, long genomic DNA fragments (20–30 kb) and by the long read lengths obtained (the N50 of the read lengths was ~16 kb). Advances in sequencing technology, such as PacBio’s long-read technology, are poised to usher in a new era of platinum-standard reference genomes.

Philippa Borrill (a postdoc in the Uauy lab, John Innes Centre, UK) described an expression visualization and integration platform (expVIP) that has been used to develop and analyze a hexaploid wheat gene-expression atlas comprising 418 diverse RNA-seq samples. This platform was crucial for integrating layers of next-generation sequencing data to reveal the genetic architecture regulating nutrient remobilization and senescence in wheat, and to prioritize targets of the senescence-controlling wheat TF NO APICAL MERISTEM b1 (NAMb1) for future study. Multiple advances such as those described above will facilitate future molecular genetic studies in crops that have large complex genomes, such as wheat and sugarcane.

## Disruptive concepts

Scientific progress is driven not only by disruptive technologies but also by disruptive concepts and out-of-the-box thinking. Several talks touched on this area. For example, Brian Gregory (University of Pennsylvania, USA) presented work from his and other labs, which aims to determine the global landscape of RNA secondary structures and RNA–protein interactions. Besides the well-known structures of non-coding RNAs, he showed how all RNAs, including mRNAs, have secondary structures whose particular conformations might influence transcript function. In addition, he discussed the potential importance of N^6^-methyladenosine (m^6^A) modification for mRNA structural integrity, which is essential for transcriptome function and homeostasis. The loss of m^6^A results in a global increase of mRNA secondary structure around start and stop codons, and many m^6^A modified transcripts are defense related. Gregory mentioned that there are 152 additional types of RNA modifications, whose functional roles remain unknown. He described a new method his lab has developed called interaction profile sequencing (PIP-seq), which allows the identification of RNA–protein interaction sites as well as proteins interacting with specific RNA structures or modifications.

Another interesting concept was that of long-distance transcriptional regulatory elements in plants, presented by Michael Dorrity (a graduate student in the Queitsch lab, University of Washington, USA). Dorrity used self-transcribing active regulatory region sequencing (STARR-seq) to rapidly identify and quantify plant enhancer elements in full-genome libraries. Using this method on *Arabidopsis thaliana*, he identified numerous elements that were enriched in intergenic and transposon sequences. Traditionally, plants are not known to harbor many long-distance enhancer elements, but Dorrity's study shows that there may be a wealth of long-distance regulatory elements in plants.

Seung Rhee (Carnegie Institution for Science, USA) described recent work to identify novel transcriptional regulators in eukaryotes. Rhee mentioned that despite unprecedented progress in genome sequencing in the past 20 years, all sequenced genomes harbor 25–75 % of protein-encoding genes whose molecular functions remain unknown. Rhee described a computational method that does not rely on sequence homology to identify new transcriptional regulators, as well as its application to several model organisms such as *A. thaliana*, *Drosophila melanogaster*, and *Homo sapiens*. Initial results include an in-depth characterization of a novel regulator that controls organ size in plants. Approaches like this could tackle the problem of assigning molecular functions to the ‘dark matter’ of genomes, which remains a problem even for intensively studied model organisms.

## New paths for harnessing crop genome diversity

Several speakers highlighted the pace with which some of that ‘dark matter’ in large crop genomes is becoming illuminated. Cristobal Uauy (John Innes Centre, UK) demonstrated the power of a genome-enabled phenotyping and sequencing platform for hexaploid bread wheat. By combining analysis of a large TILLING population with exon-capture approaches, Uauy’s team and consortium partners aim to identify wheat homoeologs that contribute to important yield traits such as grain weight.

The complexity of crop genomes is, in part, due to the expansion and diversification of disease *Resistance* (*R*) genes, which encode receptors that recognize various infectious pathogens. In the past, attempts to improve resistance relied largely on the painstaking introduction of major-effect *R* genes into high-yielding varieties for use in agriculture. Efficient capture of new, valuable resistance traits across crop germplasm is now achievable, as shown by Thomas Kroj (Institut National de la Recherche Agronomique (INRA) Montpellier, France) and Beat Keller (University of Zurich, Switzerland). Kroj highlighted the discovery of co-acting *R* gene pairs in plant genomes, which have integrated additional ‘decoy’ domains to effectively betray pathogen attack. Keller described efforts to harness the natural diversity of wheat and maize *R* gene complements against damaging fungal diseases. This is providing fundamental insights to how *R* genes evolve and functionally diversify. Information from natural allelic variants of the wheat *Pm3* powdery mildew *R* gene is enabling Keller’s team to engineer altered receptor modules and combine useful new crop disease resistance traits for robust resistance in the field.

## Seeing is believing

A prominent thread in several talks was the value of high-throughput phenotyping in model and crop plants. This is enabling the characterization of developmental processes at many scales and is uncovering the genetic variation underlying potentially useful traits. Malia Gehan (a postdoc in the Mockler lab, Donald Danforth Plant Science Center, USA) described the utility of an open-source image analysis software suite called PlantCV that she and colleagues have developed, which enables the quantification of seedling growth (biomass) and photosynthetic performance over time. Using this software and a high-throughput phenotyping platform, the Mockler group is beginning to identify natural genetic variants in the model grass species *Brachypodium distachyon*, which have altered responses to abiotic stresses such as heat and drought. Christopher Topp (also at Danforth) has focused on developing imaging tools to quantify root architectural and physiological traits in maize, and to measure the degree of plasticity in root growth dynamics in response to changing nutrient availability. Topp and a team of engineers are developing an imaging facility to measure the root behavior of maize genetic variants in the field.

These and other studies described at the meeting emphasize the multidisciplinary nature of genome-enabled quantitative biology to harness new phenotypic variation and genetic epistasis for crop improvement. Important next steps will be to determine whether the variation in growth habits, biomass, or stress tolerance observed in controlled environments contributes to bioproductivity and fitness in the field. An important cautionary tale was presented by Thomas Mitchell-Olds (Duke University, USA) who described his study of allelic variation at a biochemical defense pathway locus that underlies tolerance of *Boechera stricta* plants against insect herbivore damage. Although clearly measurable in a large natural *B. stricta* population, the fitness contribution and heritability of allelic variation at this locus is very small. This rare example of ‘real world’ ecological genetics and the effect of genotypic variation on fitness in nature emphasizes the need to measure crop productivity traits under field conditions.

## Towards predictive modeling

Descriptions of state-of-the-art, high-throughput technologies and genome biology were complemented nicely by presentations on genetic, molecular, and spatial dissections of biological processes in model and crop species. In *A. thaliana* root development, crucial transcriptional feed-forward and feed-back loops remain to be discovered, as demonstrated by Jazz Dickinson (a postdoc in the Benfey Lab, Duke University, USA) who is combining genetics, synthetic biology, and two-photon selective plane illumination microscopy (2P-SPIM) approaches to dissect spatially and temporally relevant processes in asymmetric root cell division.

George Coupland (Max Planck Institute for Plant Breeding Research (MPIPZ), Germany) outlined his group’s progress in defining the transcriptional network underlying seasonal control of flowering in *A. thaliana*. He also described variation in the network topology between annual flowering *A. thaliana* and perennial relatives such as *Arabis alpina*. Coupland identified an age-related signaling circuit that influences the ability of plants to initiate flowering. In another study, Matthias Berens (a graduate student in the Tsuda lab at MPIPZ) uncovered an effect of leaf age on hormonal decision-making in growth responses to environmental stress.

A neat combination of genetics, synthetic biology, and imaging by Ykä Helariutta (John Innes Centre, UK) is providing new insights into how early organizing centers control vascular development in *A. thaliana*. The interplay of hormone pathways regulates the sorting of cambial cells into high-order vascular tissues and secondary vascular expansion, as observed most dramatically in trees. Helariutta’s team is using knowledge gained from his analysis of *A. thaliana* to characterize the genetic basis of vascular architecture variation and adaptation in birch trees.

In another potentially exciting step forward, Kun Huang (a postdoc in the Meyers lab, University of Delaware, USA) is developing in vivo sensors to track the biogenesis and movement of small RNAs in plants in response to developmental or environmental stimuli. For this, Huang described a modified ‘Spinach’ fluorescent RNA aptamer-based detection system (FastMiR, fluorescent aptamer sensor for tracking miRNA), which works in vitro but not yet in vivo other than in Chinese Hamster Ovary cells. She described a new system using a biliverdin-binding fluorescent RNA aptamer to detect small RNAs in vivo.

## Conclusions

Here we illustrate, using a few examples, some of the exciting new directions in plant biology presented at the meeting. These endeavors leverage today’s technological revolutions in genome sequencing and imaging and will undoubtedly impact fields beyond plant biology. We, along with David Jackson (Cold Spring Harbor Laboratory, USA), are thrilled to organize the next meeting in 2017. It will be the 20^th^ anniversary of the meeting series and we anticipate a celebration of genome-enabled plant biology of the past, present, and future.

## Abbreviations

DAP-seq: DNA affinity purification sequencing
m^6^A: N^6^-methyladenosine
MPIPZ: Max Planck Institute for Plant Breeding Research
R: Resistance
TF: Transcription factor

